# Application of Honey Dressing in the Management of Postoperative Wounds, Ulcers, and Burns: A Systematic Review

**DOI:** 10.7759/cureus.106501

**Published:** 2026-04-06

**Authors:** Martin Schils, Clément Duveau, Grégory Van Doornick, Ibrahim Cherry, Hamza Tazi, Marta Misani

**Affiliations:** 1 Department of Plastic and Reconstructive Surgery, Université Libre de Bruxelles, Brussels, BEL; 2 Department of Plastic and Reconstructive Surgery, Hopital de Mons, Mons, BEL

**Keywords:** a systematic review, burn, honey, surgical wounds, ulcers

## Abstract

Honey has served as a therapeutic agent for wound treatment across diverse cultures throughout history. Despite its extensive application, the evidence substantiating the efficacy of honey in wound treatment remains unclear. The primary objective of this systematic review was to assess the existing evidence and discern the role of honey in modern wound care, specifically targeting burns, ulcers, and surgical wounds. Following the Preferred Reporting Items for Systematic Reviews and Meta-Analyses (PRISMA) guidelines, a literature search was performed across PubMed, Embase, Scopus, Cochrane Central Register of Controlled Trials (CENTRAL), Cumulative Index to Nursing and Allied Health Literature (CINAHL), Web of Science, and Google Scholar databases, identifying 22 randomized clinical trial studies deemed eligible for inclusion. Across all three categories of wounds, honey has been identified as a dressing that shortens wound healing. This effect was especially observed in burns. Furthermore, both surgical wounds and burns exhibit evidence of the antibacterial effect of honey. The anti-inflammatory effect is distinctly evident in the realm of surgical wounds. Beyond these effects, honey is acknowledged for its potential deodorizing, debridement, and, notably, wound pain-reducing properties. However, it is essential to note that the evidentiary support for these attributes is relatively limited. Burns emerge as the lesions where honey demonstrates its utmost effectiveness, likely related to a combination of antibacterial and healing-promoting properties. If confronted with escalating antibiotic resistance, the proactive utilization of honey to mitigate wound infections presents itself as a plausible and strategic option. A potential area of particular interest for future studies could be the exploration of the aesthetic effects of honey on surgical wounds.

## Introduction and background

Honey, celebrated for its remarkable healing properties, has served as a therapeutic agent for wound treatment across diverse cultures throughout history. This age-old practice can be traced back to ancient times, with evidence of its utilization discovered in the tomb of King Tutankhamen from the 14th century BC in Egyptian civilization. Additionally, the Edwin Smith papyrus, dating between 2600 and 2200 BC, attests to the medicinal application of honey. Beyond Egypt, both ancient Greeks and Romans incorporated honey, often in combination with vegetable or animal fats, to formulate various ointments aimed at preventing wound infections [1,2]. The advent of modern medicine led to a decline in the clinical utilization of honey. However, with the rise of antibiotic resistance and a growing awareness favoring natural remedies, interest in the antimicrobial and healing properties of honey has experienced a resurgence in recent years [3].

This naturally occurring substance, crafted by various bee species from plant nectar, manifests as a sweet, viscous, hypersaturated solution primarily comprised of sugar (75-79%) and water (20%). Beyond this fundamental composition, honey encompasses a multitude of beneficial elements, including proteins, B-complex vitamins, minerals, and antioxidants such as flavonoids, ascorbic acid, catalase, and selenium [4]. Organic acids, responsible for its acidity, constitute 0.57% of its composition, while major enzymes like invertase, amylase, and glucose oxidase are present at significant levels [5]. Honey transcends its simple sweetness to unveil itself as a biologically rich dressing, abundant in bioactive components, with the potential to enhance the wound-healing process [6]. Although primarily acknowledged for its antibacterial properties, honey extends beyond this attribute, as additional benefits have been ascribed to it. Research has demonstrated its anti-inflammatory characteristics, ability to debride and eliminate necrosis, neutralize undesirable odors, and promote tissue growth [7-9].

In contemporary clinical practice, the predominant variety of honey employed is Manuka honey, which originates from the *Leptospermum scoparium* tree, indigenous to New Zealand and Australia. This particular honey holds significance due to its distinct non-peroxide antibacterial activity, which differs from the hydrogen peroxide-based activity characteristic of many other honey types [10]. Medihoney is a commercially available, standardized medical-grade product derived primarily from Manuka honey. It is a blend of honey sourced from Australia and New Zealand, from the *Leptospermum* species, and showcases antibacterial activity equivalent to a phenolic acid strength of at least 18, currently positioning it as the most potent antibacterial medical honey available on the market [11].

Despite its extensive application, the evidence substantiating the efficacy of honey in wound treatment remains unclear, with many physiological effects described without a clear determination of their actual relevance [7,12]. Importantly, the composition and properties of honey vary considerably depending on geographical origin, floral source, and processing methods, which adds to the uncertainty regarding its clinical role as a standardized therapeutic intervention. While several reviews have examined the healing potential of honey, none have simultaneously assessed the broader range of therapeutic parameters, including antibacterial, anti-inflammatory, debridement, deodorization, and pain reduction effects across different wound types. This review focuses on three common types of wounds treated with honey: surgical wounds, ulcers, and burns. By conducting this review, we aim to offer healthcare professionals a more comprehensive insight into the appropriateness and genuine effectiveness of honey, considering the specific nature of different wound types.

## Review

Methodology

Research Protocol

In accordance with the Preferred Reporting Items for Systematic Reviews and Meta-Analyses (PRISMA) guidelines, a systematic review of the literature was conducted with a search of the PubMed, Embase, Scopus, Cochrane Central Register of Controlled Trials (CENTRAL), Cumulative Index to Nursing and Allied Health Literature (CINAHL), Web of Science, and Google Scholar databases until December 2023. The search strategy combined the keyword "honey" with the following terms: "burn", "chronic wounds", "ulcers", and "surgical wounds", using the Boolean operator AND. Both broad ("chronic wounds") and more specific ("ulcers") terms were included to ensure comprehensive retrieval of relevant studies. The search filters were combined into the following main strategy: ("honey"[MeSH Terms]) AND ("burn"[MeSH Terms]) OR ("honey"[MeSH Terms]) AND ("chronic wounds"[MeSH Terms]) OR ("honey"[MeSH Terms]) AND ("ulcers"[MeSH Terms]) OR ("honey"[MeSH Terms]) AND ("surgical wounds"[MeSH Terms]).

The determination of eligibility underwent independent assessments by two authors (SM and DC). Any discrepancies that arose were addressed with the involvement of a third author (MM), leading to the attainment of a unanimous consensus.

The criteria for inclusion in the studies encompassed research focused on treating burns, ulcers, and/or surgical wounds, in which the procedure involves a honey-based treatment, conducted as randomized controlled trials (RCTs), and available in either English or French. Exclusion criteria encompassed studies that did not address at least one of the six specified parameters detailed in the data collection process, as well as those concentrating on wound types other than burns, ulcers, or surgical wounds, and also those carried out on tissues derived from animals. Reports that could not be obtained in full text were excluded prior to eligibility assessment. There were no limitations on publication year, authors, or participating institutions.

Given the substantial clinical heterogeneity across the included studies, in terms of honey type, comparator dressings, wound characteristics, outcome definitions, and measurement methods, a meta-analysis was not considered feasible. The variability in the parameters assessed and the reporting formats used across studies precluded meaningful statistical pooling of results. Consequently, a narrative synthesis approach was adopted to summarize and interpret the findings.

Data Collection Process

The analysis involved a thorough examination of the study designs, such as the sample size, the specific honey dressing employed, and the type of dressing applied to the control group within each RCT. To assess the impact of honey on the different types of wounds, the two authors extracted the following six parameters. These parameters included: (i) Healing, defined as a documented reduction in wound size, shorter time to complete wound closure, or accelerated epithelialization rate compared to the control group; (ii) Antibacterial effect, defined as a documented reduction or eradication of bacterial colonization, assessed through microbiological culture results or clinical signs of infection; (iii) Anti-inflammatory effect, defined as a decrease in clinical markers of inflammation such as redness/erythema, edema, or exudate volume; (iv) Debridement effect, defined as a documented reduction in necrotic tissue or slough; (v) Pain reduction, defined as a decrease in patient-reported pain scores using scales employed in the individual studies; and (vi) Deodorization, defined as a clinically reported reduction in wound-associated malodor.

For each study and each parameter, findings were summarized using a semi-quantitative scoring system: "++" indicates a statistically significant difference in favor of honey (p<0.05); "+" indicates a difference in favor of honey that did not reach statistical significance; "0" indicates no difference between groups; "−" indicates a difference to the disadvantage of honey without statistical significance; and "NR" indicates that the parameter was not reported in the study. This scoring system was designed to provide a visual overview of the direction and strength of findings across heterogeneous studies and is not intended for quantitative pooling.

Risk of Bias

The Cochrane Risk of Bias 2 (RoB 2) tool was utilized to assess the risk of bias in the RCTs, following the official templates provided by the Cochrane Group [13]. Two reviewers (SM, DC) independently completed the assessment, evaluating bias across five domains: D1, randomization process; D2, deviations from intended interventions; D3, missing outcome data; D4, measurement of the outcome; and D5, selection of the reported result. Each domain was rated as indicating a low, some concerns, or high risk of bias, and an overall judgment was subsequently assigned to each study. Discrepancies between reviewers were resolved through discussion with a third author (MM). A summary figure and individual study assessments of risk of bias were then generated.

Results

**Figure 1:**
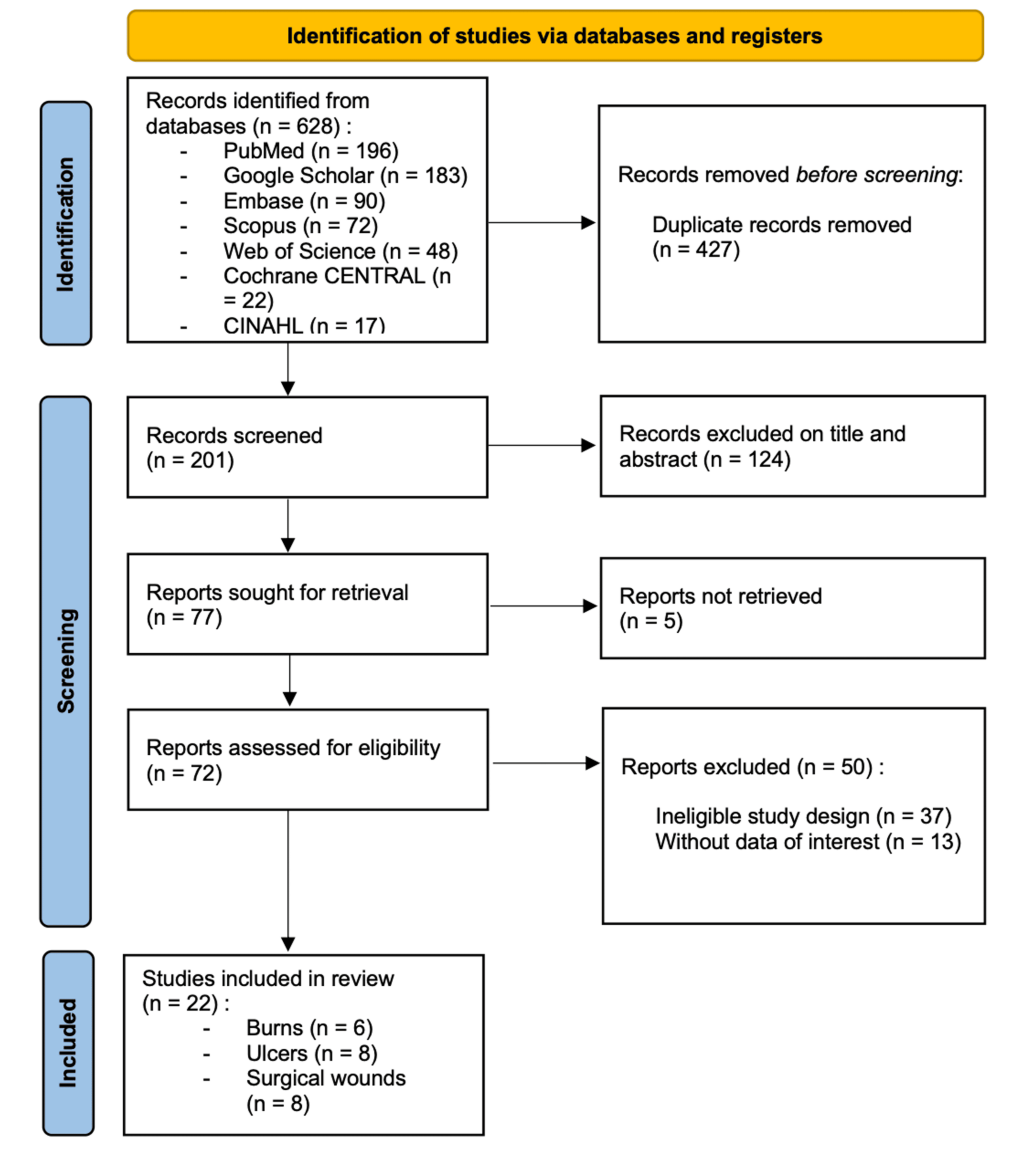
PRISMA flow chart for study selection PRISMA: Preferred Reporting Items for Systematic Reviews and Meta-Analyses

The search equation yielded 628 articles across all databases searched. After removing 427 duplicates, 201 records were screened, of which 124 were excluded based on titles and abstracts. Of the remaining 77 reports sought for retrieval, five could not be obtained in full text. Consequently, 72 reports were assessed for eligibility. Following full-text assessment, 50 articles were excluded for the following reasons: ineligible study design (n = 37) and absence of data of interest (n = 13). Ultimately, 22 studies were included in the review, of which six were centered on burns, eight on ulcers, and eight on surgical wounds (Figure 1).

Table 1 highlights significant heterogeneity in both the choice of honey and the type of dressing employed in the control groups. While some semblance exists in the choice of dressing based on the nature of the wound, such as the consistent use of silver sulfadiazine in four out of the six studies related to burns, the outcomes across different dressing types show considerable variation.

**Table 1:** Study design classified by wound type

Wound type	Study	Sample size	Therapy methods for Honey (H) and Control (C) groups
Burns	Baghel et al. [14]	n = 78: H = 37; C = 41	H: undiluted, pure; C: silver sulphadiazine
	Malik et al. [15]	n = 150 (patients with the same type of burn at two different sites)	H: Langnese; C: silver sulphadiazine
	Subrahmanyam [16]	n = 104: H = 52; C = 52	H: Langnese; C: silver sulphadiazine
	Subrahmanyam [17]	n = 92: H = 46; C = 46	H: unprocessed, undiluted; C: polyurethane film (OpSite1)
	Subrahmanyam [18]	n = 100: H = 50; C = 50	H: pure, unprocessed, undiluted (Indian hive bee); C: boiled potato peel
	Subrahmanyam [19]	n = 50: H = 25; C = 25	H: pure, unprocessed, undiluted; C: silver sulphadiazine
Ulcers	Gethin and Cowman [20]	n = 108: H = 54; C = 54	H: Manuka (Comvita), UMF 18+; C: hydrogel (IntraSite gel)
	Gethin and Cowman [21]	n = 108: H = 54; C = 54	H: Manuka (Comvita), UMF 18+; C: hydrogel (IntraSite gel)
	Jull et al. [22]	n = 368: H = 187; C = 181	H: Manuka (ApiNateTM, Comvita), UMF 12+; C: standard dressings (alginate, foam, hydrofibre, hydrocolloid, hydrogel, non-adherent, iodine or silver)
	Shukrimi et al. [23]	n = 30	H: clean, non-sterile, pure; C: povidone-iodine 10%
	Yapucu Güneş and Eşer [24]	n = 50: H = 15; C = 11 (in 26 patients)	H: sterile, unprocessed (raw, natural, organic, unpasteurized) from one source, MIC = 3.8%; C: ethoxy-diaminoacridine plus nitrofurazone dressings
	Oluwatosin et al. [25]	n = 50	H: unprocessed and undiluted; C: phenytoin/honey mixture and phenytoin
	Al Saeed [26]	n = 118: H = 59; C = 59	H: Manuka and conventional treatment; C: conventional treatment alone
	Suryaprakash et al. [27]	n = 90: H = 45; C = 45	H: dressing soaked with honey; C: povidone XI iodine dressings
Surgical wounds	Ahmed et al. [28]	n = 60	H: HoneySoft1 (=Chilian medicinal multifloral); C: none
	Kumarasamy et al. [29]	n = 32	H: Tualang honey; C: Triamcinolone
	Altaweel et al. [30]	n = 80	H: natural honey; C: none
	Mphande et al. [31]	n = 40: H = 22; C = 18	H: honey from Malawi; C: sugar from Malawi
	Goharshenasan et al. [32]	n = 52: H = 52; C = 52	H: honey from Uraman area; C: Vaseline
	Nikpour et al. [33]	n = 38: H = 38; C = 37	H: natural honey; C: none
	Malhotra et al. [34]	n = 46: H = 46; C = 46	H: Manuka; C: Vaseline
	Anyanechi and Saheeb[35]	n = 72: H = 36; C = 36	H: Obudu honey; C: dilute hydrogen peroxide alternated with normal saline

Burns

Six RCTs explored the effect of honey dressings in burns [14-19]. The included studies predominantly assessed superficial and partial-thickness burns (first- and second-degree burns). These studies showed significant findings favoring the repercussion of honey in reducing the time required for complete wound healing. Regarding the antibacterial properties, five out of six studies reported results in favor of honey dressings [12-18], with four showing a statistically significant difference [14,16-18]. For the remaining four parameters analyzed, including debridement effect, anti-inflammatory properties, and the reduction of odor and pain, multiple studies addressed these dimensions. While certain studies indicated superior results of honey compared to the control group on these parameters, none yielded statistically significant results (Table 2).

**Table 2:** Results for the six parameters selected according to wound type ++ : significant difference in favour of honey; + : difference in favour of honey but not significant; 0 : No difference; -  : difference to the disadvantage of honey but not significant; ;NR : not reported

Wound type	Study	Healing	Antibacterial effect	Anti-inflammatory effect	Debridement	Odour reduction	Pain reduction
Burns	Baghel et al. [14]	++	++	NR	+	NR	NR
	Malik et al. [15]	++	+	NR	NR	NR	NR
	Subrahmanyam [16]	++	++	NR	NR	NR	+
	Subrahmanyam [17]	++	++	NR	NR	NR	NR
	Subrahmanyam [18]	++	++	NR	NR	NR	+
	Subrahmanyam [19]	++	NR	+	+	NR	NR
Ulcers	Gethin and Cowman [20]	NR	+	NR	NR	NR	++
	Gethin and Cowman [21]	++	NR	NR	+	NR	NR
	Jull et al. [22]	0	0	NR	NR	NR	NR
	Shukrimi et al. [23]	0	0	+	NR	+	NR
	Yapucu Güneş and Eşer [24]	++	NR	NR	NR	NR	NR
	Oluwatosin et al. [25]	-	NR	NR	NR	NR	0
	Al Saeed [26]	++	+	+	+	NR	NR
	Suryaprakash et al. [27]	++	NR	NR	+	NR	-
Surgical Wounds	Ahmed et al. [28]	+	+	+	+	+	-
	Kumarasamy et al. [29]	+	+	NR	+	NR	+
	Altaweel et al. [30]	++	++	+	+	++	NR
	Mphande et al. [31]	+	+	NR	NR	NR	+
	Goharshenasan et al. [32]	++	+	++	NR	NR	NR
	Nikpour et al. [33]	++	++	++	NR	NR	NR
	Malhotra et al. [34]	+	NR	NR	NR	NR	NR
	Anyanechi and Saheeb [35]	+	0	+	NR	NR	++

Ulcers

Eight studies investigated the effects of honey on ulcers [20-27]. The types of ulcers included in this review were venous leg ulcers, diabetic foot ulcers, and pressure ulcers. The included studies did not use a uniform definition of chronicity based on duration, but generally referred to wounds that had failed to progress through normal healing stages within an expected timeframe. The predominant attribute of honey emphasized was its established capacity to foster tissue regeneration, with four studies reporting a statistically significant reduction in wound size [21,24,26,27], while two studies did not observe a statistically significant difference [22,23]. One study reported a trend against honey for healing [25], and one study did not report this outcome [20]. Among the findings, four studies documented the antibacterial effect of honey [20,22,23,26], but only two of them demonstrated results in favor of honey, albeit without reaching statistical significance [20,26]. Regarding the anti-inflammatory, deodorizing, debriding, and pain-relieving properties of honey in ulcers, each of these aspects is supported by at least one study [20,21,23,26,27]. However, only the reduction in pain reached statistical significance [20]. It is noteworthy that one study diverged from this consensus, attributing post-application pain to the use of honey on ulcers, though this observation did not reach statistical significance [27].

Surgical Wounds

Our investigation unveiled eight RCTs assessing the impact of honey on post-operative surgical wounds [28-35]. The findings from these studies show an overall trend in favor of honey, with three studies reporting statistically significant outcomes [30,32,33]. The antibacterial effect and the anti-inflammatory effect are also in favor of honey. Two studies managed to statistically prove their conclusions concerning each of these properties ([30,33] and [32,33], respectively). Regarding pain reduction, results remain less conclusive [28,29,31,35], while one study demonstrated a statistically significant reduction in malodor [30]. While outcomes relating to the debridement effect lean in favor of honey, the trend suggests an advantage without reaching statistical significance [28-30].

Risk of Bias

The RoB-2 tool was employed to assess all 22 studies across five domains, aiming to discern potential biases in methodology and outcomes (Table 3).

**Table 3:** Risk of Bias (RoB-2) assessment of included studies according to wound type S = some concerns; L = low risk of bias; H = high risk of bias. Risk of bias was assessed using the Cochrane Risk-of-Bias 2 (RoB-2) tool across five domains: randomisation process, deviations from intended interventions, missing outcome data, measurement of the outcome, and selection of the reported result.

Wound type	Study	Randomisation process	Deviations from intended interventions	Missing outcome data	Measurement of the outcome	Selection of the reported result	Overall risk of bias
Burns	Baghel et al. [14]	S	L	L	S	S	S
	Malik et al. [15]	S	L	L	S	S	S
	Subrahmanyam [16]	S	H	L	S	S	S
	Subrahmanyam [17]	S	H	L	S	S	S
	Subrahmanyam [18]	S	H	S	S	S	S
	Subrahmanyam [19]	S	H	L	S	S	S
Ulcers	Gethin and Cowman [20]	S	H	S	S	L	H
	Gethin and Cowman [21]	S	H	S	S	L	H
	Jull et al. [22]	L	H	S	S	L	H
	Shukrimi et al. [23]	S	H	L	L	S	S
	Yapucu Güneş and Eşer [24]	S	H	H	S	L	H
	Oluwatosin et al. [25]	S	H	S	S	S	H
	Al Saeed [26]	L	L	L	L	S	L
	Suryaprakash et al. [27]	S	H	L	S	S	S
Surgical wounds	Ahmed et al. [28]	L	L	L	L	S	L
	Kumarasamy et al. [29]	S	H	L	S	S	S
	Altaweel et al. [30]	L	S	L	S	L	S
	Mphande et al. [31]	S	H	L	S	S	S
	Goharshenasan et al. [32]	S	L	L	S	S	S
	Nikpour et al. [33]	L	L	L	L	S	L
	Malhotra et al. [34]	S	L	S	L	S	S
	Anyanechi and Saheeb [35]	S	H	L	S	S	S

The study by Malik et al. was assessed as having some concerns regarding risk of bias, particularly related to allocation concealment [15]. Notably, only Malik et al.'s study explicitly detailed patient and nursing staff blinding; other studies managed potential performance bias by employing objective primary outcome measures. Most trials exhibited an uncertain risk of bias regarding the blinding of participants and outcome assessors. Nevertheless, five studies evaluated the primary outcome using a subjectively interpreted measure of 'complete healing,' which led to a heightened risk of bias in outcome assessment [16-19,30].

Regarding incomplete outcome data, none of the studies included experienced any loss to follow-up. Trials were considered to have a low risk of bias concerning 'selective outcome reporting' when the outcomes specified in the methods section were also reported in the results. Except for two trials that did not have a predetermined plan for data analysis and reporting [23,25], posing challenges in identifying reporting bias, all other trials were deemed to have a low risk of bias.

Regarding other potential biases, the emphasis was placed on the initial comparability between honey and basic treatment, contingent upon the wound type. Nevertheless, three studies concerning surgical wounds failed to disclose any treatment utilization in the control group [28,29,33]; all other trials documented comparable baseline characteristics between the intervention and control groups.

Discussion

Healing

To our knowledge, it is the first systematic review delving into the examination of six distinct effects attributed to honey dressing across three diverse wound classifications. Yilmaz and Aygin undertook a similar systematic review, focusing on the healing disparities among surgical wounds, ulcers, and burns [36]. However, their evaluation was confined solely to the efficacy of honey in promoting healing, neglecting the assessment of the remaining five parameters.

In line with the findings of Yilmaz and Aygin [36], our review yields comparable outcomes regarding the healing potential of honey across all three wound types. Our results particularly underscore the efficacy of honey in treating burns, where all six studies demonstrated statistically significant effects compared to the control group [14-19]. Among these, four specifically compared honey to silver sulfadiazine, currently acknowledged as the global standard for burn care [14-16,19]. These findings corroborate the conclusions drawn in previously conducted systematic reviews and meta-analyses comparing silver-based and honey-based dressings for burns [37-39].

While the results are particularly compelling in the context of burns, the outcomes for ulcers and surgical wounds, while somewhat less pronounced, still hold considerable promise. As an illustration, Al Saeed's 2013 study demonstrated that the utilization of Manuka honey dressing led to a substantial healing rate of 61.3%, in sharp contrast to the 11.5% observed in the control group [26]. Additional insights from external literature, such as the study by Robson et al., have reported a reduction in healing time, with honey treatment taking 100 days compared to 140 days with a hydrogel [40]. The expedited healing time with honey can be attributed to an accelerated epithelialization process in its presence [15,17] and its stimulating effect on the formation of healthy granulation tissue [16,18].

Antibacterial Properties

The robust evidence supporting the antibacterial properties of honey dressing is most pronounced in studies focusing on burns. The evidence supporting the antibacterial efficacy of honey for ulcers and surgical wounds is considered moderate to weak.

The following mechanisms have been proposed in the preclinical literature to explain the antibacterial effectiveness of honey, although it should be noted that these mechanisms were not directly assessed in the clinical trials included in this review. Primary among them is the production of hydrogen peroxide during the dilution of honey with wound exudate [6]. Synthesized by the enzyme glucose oxidase, this hydrogen peroxide exerts a dual impact by activating neutrophils, thereby reinforcing the inflammatory response, and concurrently acting directly as a bactericidal agent, causing irreversible damage to bacterial cell membranes, proteins, enzymes, and DNA [40].

Additionally, based on in vitro evidence, the flavonoids present in honey play a crucial role by directly inhibiting phagocytosis. These antioxidants serve as key players in preventing the formation of superoxide free radicals, protecting tissues against potential damage [13].

Finally, the viscosity, hyperosmolarity, and acidity of honey, characterized by a pH between 3.2 and 4.5, impede the growth of microorganisms, given that the optimal pH for the majority of these organisms falls between 7.2 and 7.4 [41].

Studies have demonstrated the effectiveness of honey dressings against various bacteria, including *Escherichia coli*, *Pseudomonas aeruginosa*, *Staphylococcus aureus*, *Acinetobacter*, and *Stenotrophomonas*. Notably, honey has exhibited efficacy against antibiotic-resistant bacterial strains, such as methicillin-resistant *S. aureus* and vancomycin-resistant *Enterococcus* [42,43]. The combination of these diverse mechanisms bestows honey with an extensive antibacterial profile, rendering it effective in managing infections in wounds.

In considering future research avenues, it would be intriguing to delve into the therapeutic potential of combining antibacterial agents with honey, especially for treating wound types where the efficacy of honey alone hasn't been demonstrated, such as ulcers and surgical wounds. A systematic review on this topic indicates a heightened efficacy when honey is combined with specific therapeutic agents, surpassing the effectiveness of honey used in isolation. Exploring this synergistic approach could unveil novel insights into wound treatment strategies [44].

Anti-Inflammatory Properties

The exploration of the anti-inflammatory properties of honey has been relatively limited in the examined studies, except in surgical wounds, where five studies reported favorable outcomes with honey dressings [28,30,32,33,35], of which two reached statistical significance [32,33]. Based on preclinical evidence, the anti-inflammatory effect of honey is attributed to the presence of plant phenolic compounds, which inhibit macrophage phagocytosis, thereby contributing to the attenuation of inflammatory processes [45]. However, this mechanism has been demonstrated primarily in laboratory settings and was not directly assessed in the clinical trials under review. It is noteworthy that, at present, the anti-inflammatory effect has primarily been demonstrated clinically in the context of surgical wounds. More comprehensive studies across other wound types would be instrumental in confirming this parameter on a broader scale.

Deodorization, Debridement, and Pain Reduction Properties

The evidence supporting honey's deodorization, debridement, and pain reduction properties in the context of wounds is rather limited [46-48]. Our review highlighted that few studies have managed to demonstrate truly significant results in these domains. The ability of honey to facilitate wound debridement is theoretically explained in the literature through the action of protease enzymes and its potential to initiate autolytic debridement [49,50]. However, no study has provided statistically significant results for this parameter, leaving this physiological explanation in a theoretical realm. Indeed, in 2020, a systematic review of in vivo animal studies unveiled evidence that the active biomolecules present in honey play a significant role in enhancing the process of autolytic debridement and promoting the formation of healthy tissue granulation [51].

Concerning the last two parameters-the ability of honey to neutralize bad odors and reduce the perception of pain-they are more restricted in terms of physiological explanation. The understanding of these two mechanisms remains incomplete, even though a few studies have furnished significant evidence.

Other Properties in Surgical Wounds

Beyond the analysis of the previous properties, it becomes imperative to consider additional criteria that are more specific to the post-operative phase in order to comprehensively assess the impact of honey dressings. In this regard, two studies have captured our attention: Goharshenasan et al.'s study in 2016 [32] and Nikpour et al.'s study in 2014 [33]. These studies have successfully demonstrated a significant reduction in complications, such as cast reopening, coupled with the achievement of improved aesthetic outcomes. Delving further into the exploration of these two parameters could prove to be particularly enlightening in advancing our understanding of the effects of honey treatment.

Strengths and Limitations of this Review

The strength of this review lies in its ability to offer a comprehensive perspective on the utilization of honey in three distinct categories of wound management, while also underscoring gaps in the findings and methodologies of the included studies.

However, several limitations should be acknowledged. First, a meta-analysis was not performed due to the substantial clinical heterogeneity across studies in terms of honey type, comparator dressings, wound characteristics, and outcome measurement methods. While some subgroups-particularly burn studies comparing honey to silver sulfadiazine-showed greater homogeneity, the overall diversity of outcomes and reporting formats precluded rigorous statistical pooling.

Second, the considerable heterogeneity of the included studies is reflected in the wide variability of honey types used (Manuka, raw, unprocessed, local varieties), with sterilization and processing methods, application protocols, dressing frequency, and honey composition often unspecified. This lack of standardization limits the reproducibility and generalizability of the findings and weakens conclusions regarding "honey" as a unified intervention. Future studies should systematically report the type, source, processing, and composition of honey used to enable more meaningful comparisons.

Third, the semi-quantitative scoring system employed (++/+/0/−/NR) was designed as a visual summary tool and is not a validated scale. The heterogeneity of outcome measures across studies, including different pain scales, varying definitions of "complete healing," and diverse microbiological methods, made a standardized quantitative approach impractical. This reinforces the rationale for a narrative synthesis but also limits the precision of our conclusions.

Finally, caution should be exercised when interpreting the results on burns. Four of the six burn studies were conducted by a single researcher, Subrahmanyam [16-19], raising concerns about potential author bias and methodological duplication, which may lead to an overestimation of treatment effects. Additionally, all burn studies used pure, undiluted honey without specifying its composition. Readers should interpret burn-related findings with particular caution, and future reviews should critically appraise the independence of study populations when multiple publications originate from the same research group.

## Conclusions

While the therapeutic benefits of honey have been acknowledged for centuries, contemporary studies consistently affirm its efficacy in the wound healing process, particularly when comparing honey-based dressings with conventional medical products currently in practice. Burns, emerge as the lesions where honey demonstrates its utmost effectiveness, likely related to a combination of antibacterial and healing-promoting properties. If confronted with escalating antibiotic resistance, the proactive utilization of honey to mitigate wound infections presents itself as a plausible and strategic option.

Beyond its notable antibacterial action, honey manifests various other properties that open avenues for promising future research. Among these, a potential area of particular interest for future studies could be the exploration of the aesthetic effects of honey on surgical wounds. This underscores the evolving landscape of the multifaceted contributions of honey to wound care and emphasizes the need for further investigations in various dimensions of its therapeutic potential.
